# Effect of multimorbidity on utilisation and out-of-pocket expenditure in Indonesia: quantile regression analysis

**DOI:** 10.1186/s12913-021-06446-9

**Published:** 2021-05-05

**Authors:** Kanya Anindya, Nawi Ng, Rifat Atun, Tiara Marthias, Yang Zhao, Barbara McPake, Alexander van Heusden, Tianxin Pan, John Tayu Lee

**Affiliations:** 1 The Nossal Institute for Global Health, Melbourne School of Population and Global Health, The University of Melbourne, Melbourne, VIC Australia; 2 School of Public Health and Community Medicine, Institute of Medicine, Sahlgrenska Academy, The University of Gothenburg, Gothenburg, Sweden; 3 Department of Global Health and Population, Harvard T.H. Chan School of Public Health, Harvard University, Boston, MA USA; 4 Department of Public Health, Faculty of Medicine, Public Health and Nursing, Universitas Gadjah Mada, Yogyakarta, Indonesia; 5 The George Institute for Global Health at Peking University Health Science Center, Beijing, China; 6 WHO Collaborating Centre on Implementation Research for Prevention and Control of Noncommunicable Diseases, Melbourne, VIC Australia; 7 Centre for Health Policy, School of Population and Global Health, The University of Melbourne, Melbourne, Australia; 8 Department of Primary Care and Public Health, School of Public Health, Imperial College London, London, UK

**Keywords:** Multimorbidity, Indonesia, Non-communicable diseases, Health service use, Out-of-pocket expenditure

## Abstract

**Background:**

Multimorbidity (the presence of two or more non-communicable diseases) is a major growing challenge for many low-income and middle-income countries (LMICs). Yet, its effects on health care costs and financial burden for patients have not been adequately studied. This study investigates the effect of multimorbidity across the different percentiles of healthcare utilisation and out-of-pocket expenditure (OOPE).

**Methods:**

We conducted a secondary data analysis of the 2014/2015 Indonesian Family Life Survey (IFLS-5), which included 13,798 respondents aged ≥40 years. Poisson regression was used to assess the association between sociodemographic characteristics and the total number of non-communicable diseases (NCDs), while multivariate logistic regression and quantile regression analysis was used to estimate the associations between multimorbidity, health service use and OOPE.

**Results:**

Overall, 20.8% of total participants had two or more NCDs in 2014/2015. The number of NCDs was associated with higher healthcare utilisation (coefficient 0.11, 95% CI 0.07–0.14 for outpatient care and coefficient 0.09 (95% CI 0.02–0.16 for inpatient care) and higher four-weekly OOPE (coefficient 27.0, 95% CI 11.4–42.7). The quantile regression results indicated that the marginal effect of having three or more NCDs on the absolute amount of four-weekly OOPE was smaller for the lower percentiles (at the 25th percentile, coefficient 1.0, 95% CI 0.5–1.5) but more pronounced for the higher percentile of out-of-pocket spending distribution (at the 90th percentile, coefficient 31.0, 95% CI 15.9–46.2).

**Conclusion:**

Multimorbidity is positively correlated with health service utilisation and OOPE and has a significant effect, especially among those in the upper tail of the utilisation/costs distribution. Health financing strategies are urgently required to meet the needs of patients with multimorbidity, particularly for vulnerable groups that have a higher level of health care utilisation.

**Supplementary Information:**

The online version contains supplementary material available at 10.1186/s12913-021-06446-9.

## Introduction

Non-communicable diseases (NCDs) are the leading cause of disease burden worldwide, contributing to approximately three-quarters of total deaths in 2017, with over 85% of premature mortality from NCDs occurring in low-income and middle-income countries (LMICs) [1]. The prevalence of multimorbidity, defined as having two or more chronic NCDs in a person, is rising with increased longevity and greater exposure to NCD risk factors in many countries [2, 3]. Despite the growing prevalence of multimorbidity in LMICs, there are very few studies on the financial burden and impoverishing effects of multimorbidity on individuals and households [2].

Indonesia, the fourth most populous country globally with a population of 270 million people in 2020 [4], has experienced rapid demographic and epidemiological transitions over the last two decades. It is estimated that the proportion of the citizens aged 65 years and over within the national population will increase from 10% in 2020 to 16% in 2035 [5]. The change in the population age structure leads to an epidemiological transition with increased prevalence of multimorbidity which poses enormous challenges for both individuals and the health system in Indonesia. Financial protection strategies are crucial in protecting household with older adults against the burden of out-of-pocket expenditures (OOPE) [6]. In 2014, the Government of Indonesia rolled out the world’s largest single-payer health insurance programme, *Jaminan Kesehatan Nasional* (JKN), which covers 84% of citizens in 2019 [7, 8]. This is the world’s largest natural experiment of the role of such a programme in improving access to healthcare services. However, OOPE accounts for a significant source of financing for the Indonesian Health System, accounting for 40% of total health expenditures in 2018 [9]. High levels of OOPE can exacerbate the burden of illness in individuals due to delayed or forgone care, place a strain on personal finances, and lead to an increased likelihood of financial catastrophe, impoverishment and worsening of social determinants of health [9, 10]. These adverse effects have greater consequences for the poor and vulnerable populations [11, 12] and contribute to further widening inequalities in health among population groups [13].

The economic impact of multimorbidity is a topic of growing research inquiry. Evidence from high-income countries (HICs) suggest that multimorbidity imposes a substantial economic burden, however, evidence in LMICs and in particular Indonesia is sparse [14–16]. Moreover, previous studies in LMICs utilised regression models, such as ordinary least squares (OLS), generalized linear model (GLM), or logistic models, that estimate the impact of multimorbidity on outcomes at the mean or population average [3, 17–19]. These regression methods often assume that regression coefficients are constant across the population, ignoring the fact that the effect of multimorbidity may vary in those with higher utilisation than those with lower utilisation. Alternative estimation strategy using quantile regression analysis has been increasingly adopted in health systems research to examine the associations between outcomes of interest and the explanatory variables across the distribution of a given dependent variable. Using a large population-based survey in Indonesia, our study aims to examine the effect of multimorbidity across the different percentiles of healthcare utilisation and OOPE.

## Methods

### Sample and data

This study used cross-sectional data, collected from 2014 to 2015, from the fifth wave of the Indonesian Family Life Survey (IFLS-5). IFLS-5 included 50,148 individuals across 16,204 households, covering 24 out of the 34 Indonesian provinces [20]. The dataset contained individuals’ and their household information, including sociodemographic characteristics, health status and history of diseases (including NCDs), and healthcare utilisation. Information about NCDs was only available for those aged 40 years and above. As such, the age group 40 years and above was the focus of the analysis. Extensive descriptions of the survey objectives and methods are available elsewhere [21]. Only respondents aged 40 years and above who had completed Book IIIB section ‘chronic disease’ were included in our analysis. Individuals with missing values for outcome variables (0.7% of the sample) were excluded. Post exclusions, the final sample totalled 13,798 respondents (sample flowchart-Additional file 1: Appendix 1). We reported this study according to the Strengthening the Reporting of Observational Studies in Epidemiology (STROBE) guidelines [22].

### Variables

#### Multimorbidity

The primary explanatory variable of interest was multimorbidity, defined as the presence of two or more chronic NCDs. A total of 14 NCDs were included in the IFLS-5: high blood pressure, diabetes, asthma, heart attack/coronary heart disease, liver disease, stroke, cancer, arthritis/rheumatism, high cholesterol, prostate illness (for male respondents), kidney disease (excluding malignancy), digestive disease, mental illness, and memory-related diseases. Respondents who answered affirmatively to the question “Has a doctor/paramedic/nurse/midwife ever told you that you had any of these conditions?” were defined as reporting an NCD.

All respondents aged 15 years and over had their blood pressure measured three times on alternate arms using Omron self-inflating sphygmomanometers by trained nurses. In our analysis, a respondent was categorised as having high blood pressure if the mean systolic blood pressure of ≥140 mmHg and/or mean diastolic blood pressure of ≥90 mmHg was measured, or the respondent self-reported as previously having been diagnosed with high blood pressure [23]. We also quantified the total number of NCDs for each respondent (0 to 13 for female, or, 14 for male respondents). Detailed definitions and categorisations are available in Additional file 1: Appendix 2.

#### Outcome variable

Health service use and four-weekly OOPE served as our primary outcome variables. Respondents were asked about the frequency of outpatient care (number of outpatient care in the last 4 weeks) and inpatient care (number of inpatient care in the last 12 months). Four-weekly OOPE was defined as the sum of direct payments associated with outpatient and/or inpatient care incurred during a four-week period, after reimbursement from health insurance. The respondents were asked: “How much did you pay out-of-pocket for outpatient care during the past four weeks?” and: “How much did you pay out-of-pocket for inpatient care in the past year?”. We calculated the average four-weekly OOPE by dividing annual OOPE for inpatient care by 13 (as the reference period of inpatient expenditure in the IFLS-5 is in the past year and a year consists of 52 weeks) and added OOPE for outpatient care. The OOPE was adjusted for inflation and was translated to 2014 International Dollars [24].

The following covariates included in this analysis were: sex (male, female), age groups (40–49, 50–59, 60–69, 70+ years), marital status (not currently married, currently married), education (no education, primary, junior high school, senior high school, tertiary), coverage of health insurance (no, yes), and respondents’ economic status (per capita expenditure for consumption) which was categorised into quintiles: q1 (lowest) to q5 (highest). Residency (rural, urban) and region of residence (Java-Bali, Sumatra, Nusa Tenggara, Kalimantan, Sulawesi) were also included as covariates. Detailed definitions are available in Additional file 1: Appendix 2.

### Statistical approach

This study assessed the effect of multimorbidity on health service utilisation and OOPE using Poisson regression, logistic regression, and quantile regression model. Poisson regression was used to assess the association between sociodemographic characteristics and the total number of NCDs. In this study, Poisson regression is more appropriate than negative binomial regression due to under-dispersion of the data (goodness of fit: *p*-value > 0.05, alpha = 0) [25]. Logistic regression analysis was used to assess the association between sociodemographic characteristics and multimorbidity.

We further assessed the effect of multimorbidity on health service use and the OOPE using a two-part model. In this model specification, a binary choice model was fitted for the probability of observing the use of outpatient/inpatient care or reported OOPE versus no visit or no OOPE using logistic regression. Conditional on a positive outcome, a quantile regression model was then fit for the positive health service utilisation and OOPE outcomes. Quantile regression was adapted over the ordinary least square (OLS) due to the highly skewed distribution of the outcome variables. This approach is less sensitive to the influence of outliers as it provides estimations of the impact of an explanatory variable along the whole distribution of outcomes variables [26, 27]. Moreover, quantile regression fit a line that minimises the sum of the absolute residuals [26, 27]. We assessed the association between multimorbidity and various percentiles of health service use/OOPE (from 10th to 90th percentiles). The coefficients at lower percentiles (e.g., 10th, 20th percentile) measure the association between multimorbidity and outcomes on those with low utilisation/OOPE, while the coefficients at upper percentiles (e.g., 70th, 90th percentiles) reflect the association on those with higher utilisation and OOPE.

Marginal effects were estimated in outcomes in relation to the number of NCDs using the following approach. First, we assessed the incremental differences in outcomes in absolute values. In the regression analysis, we included multimorbidity (categorical variable of no NCD, one NCD, two NCDs, three or more NCDs) as a dummy variable to estimate differences in outcomes as opposed to those with no NCDs (the reference group). Second, we estimated the effect of multimorbidity on the outcomes using the total number of NCDs (continuous variable) reported by an individual. We applied sampling weights to account for the complex multiple-stage design of the IFLS-5 survey. We conducted all statistical analyses using Stata 14.2 (Stata Corp., College Station, Texas).

## Results

The study sample consisted of 13,798 respondents. The sample characteristics are described in Table 1 and Additional file 1: Appendix 3. The median age was 58 years old (IQR 54–65), 51% of respondents were female, 80.32% were married, 39.36% had no education, 42.11% had health insurance coverage, and 75.71% resided in the Java-Bali region. Overall, 20.84% of respondents reported having two or more NCDs.

**Table 1:** The association between individual sociodemographic characteristics, number of NCDs, and multimorbidity

Characteristics	Total		Number of NCDs^**a**^			Any multimorbidity^b,c^		
	n	%	PR	95% CI	*p*-value	aOR	95% CI	*p*-value
**Overall**	13,798	100.0						
Age (year)								
40–49 years	5872	44.63	Ref.			Ref.		
50–59 years	3999	29.02	1.44	(1.37–1.52)	< 0.0001	1.94	(1.72–2.18)	< 0.0001
60–69 years	2210	15.46	1.77	(1.68–1.87)	< 0.0001	2.86	(2.50–3.28)	< 0.0001
70+ years	1717	10.89	1.89	(1.77–2.00)	< 0.0001	3.06	(2.60–3.60)	< 0.0001
**Gender**								
Male	6627	49.00	Ref.			Ref.		
Female	7171	51.00	1.29	(1.24–1.35)	< 0.0001	1.67	(1.51–1.84)	< 0.0001
**Marital status**								
Not currently married	2928	19.68	Ref.			Ref.		
Currently married	10,870	80.32	1.06	(1.01–1.11)	0.012	1.09	(0.97–1.23)	0.147
**Education status**								
No education	5359	39.36	Ref.			Ref.		
Primary	3237	24.79	1.07	(1.02–1.12)	0.010	1.22	(1.08–1.38)	0.002
Junior high school	1570	11.13	1.11	(1.04–1.20)	0.003	1.31	(1.11–1.54)	0.001
Senior high school	2587	17.67	1.09	(1.03–1.16)	0.006	1.27	(1.09–1.48)	0.002
Tertiary	1045	7.050	1.19	(1.10–1.29)	< 0.0001	1.60	(1.32–1.93)	< 0.0001
**Residency**								
Rural	5850	49.07	Ref.			Ref.		
Urban	7948	50.93	1.11	(1.06–1.15)	< 0.0001	1.30	(1.17–1.44)	< 0.0001
**Region**								
Java-Bali	8694	75.71	Ref.			Ref.		
Sumatra	2920	16.10	1.08	(1.03–1.13)	0.001	1.29	(1.15–1.43)	< 0.0001
Nusa Tenggara	863	2.60	0.73	(0.68–0.79)	< 0.0001	0.50	(0.41–0.63)	< 0.0001
Kalimantan	602	2.56	1.19	(1.10–1.29)	< 0.0001	1.56	(1.28–1.89)	< 0.0001
Sulawesi	719	3.03	0.99	(0.91–1.07)	0.771	1.03	(0.84–1.26)	0.763
**PCE, quartile**								
Q1 (the lowest)	2761	21.36	Ref.			Ref.		
Q2	2761	21.04	1.09	(1.02–1.15)	0.007	1.34	(1.15–1.57)	< 0.0001
Q3	2766	19.78	1.19	(1.12–1.27)	< 0.0001	1.57	(1.34–1.84)	< 0.0001
Q4	2759	19.46	1.24	(1.16–1.32)	< 0.0001	1.66	(1.42–1.94)	< 0.0001
Q5 (the highest)	2751	18.37	1.39	(1.30–1.48)	< 0.0001	2.06	(1.76–2.41)	< 0.0001
**Had any health insurance**								
No	6925	52.11	Ref.			Ref.		
Yes	6873	47.89	1.14	(1.09–1.18)	< 0.0001	1.35	(1.23–1.49)	< 0.0001

*NCDs* non-communicable diseases, *PCE* per capita expenditure

^a^Prevalence ratio (PR) was estimated using Poisson regression

^b^Adjusted Odds Ratio (aOR) was estimated using logistic regression model

^c^We defined multimorbidity if the respondents reported that they had two or more chronic conditions related to NCDs. Chronic diseases in IFLS5 included hypertension, diabetes mellitus, asthma, chronic heart diseases, mental health issue, stroke, liver diseases, cancer/malignancies, liver, arthritis, high cholesterol, prostate illness kidney diseases, digestive system diseases

Table 1 shows the association of sociodemographic characteristics, number of NCDs and the prevalence of multimorbidity. Results of the Poisson regression suggests that the number of NCDs increases substantially with age (for aged 70+ years, prevalence rate [PR] 1.89, 95% confidence interval [CI] 1.77–2.00), higher educational level (for tertiary level, PR 1.19, 95% CI 1.10–1.29), and higher socioeconomic status (for highest quantile, PR 1.39, 95% CI 1.30–1.48).

Similarly, the prevalence of multimorbidity increased with people being older. For example, participants aged 60–69 years (adjusted odds ratio [aOR] 2.86, 95% CI 2.50–3.28) and 70+ years (aOR 3.06, 95% CI 2.60–3.60) were more likely to have multimorbidity compared with participants aged 40–49 years. Female (aOR 1.67, 95% CI 1.51–1.84) were more likely to report multimorbidity than male. The prevalence of multimorbidity was greater in participants with higher educational attainment. For example, those with tertiary or higher education (aOR 1.60, 95% CI 1.32–1.93) were more likely to have multimorbidity compared with those with no education. The odds of having multimorbidity was twice as much for those in the most affluent group (fifth quantile of per capita expenditure) (aOR 2.06, 95% CI 1.76–2.41) compared with those in the lowest quantile.

Table 2 shows the proportion and frequency of health service use and OOPE across the number of NCDs. Of the total participants, 20.80% (95% CI 20.08–21.55) had at least one outpatient care in the past 4 weeks, and 4.35% (95% CI 4.01–4.69) were admitted into an inpatient facility in the past year. A greater proportion of individuals with three or more NCDs had outpatient care (44.83, 95% CI 41.64–48.02) compared with those without any NCD (13.31, 95% CI 12.026–14.39). Similarly, for inpatient care, 14.34% (95% CI 11.93–16.75) of those with three or more NCDs used inpatient services within 1 year of the survey compared with 1.83% (95% CI 1.46–2.15) for those without any NCD. Individuals with multimorbidity appear to have a higher proportion of the occurrence of OOPE (for having two NCDs 22.62, 95% CI 20.57–24.67; for having three or more NCDs 30.94, 95% CI 27.89–33.99) compared with those with no or only one NCD (10.76, 95% CI 9.79–11.72 and 15.43, 95% CI 14.36–16.50, respectively). Having three or more NCDs was observed to have a substantially higher four-weekly OOPE of Int$69.3 (95% CI $40.3–98.4) compared with Int$23.1 (95% CI $18.2–28.0) for those without any NCDs.

**Table 2:** Mean of health service use and expenditure in 2014 by number of NCD(s)

Outcomes	All participants	No NCD	One NCD	Two NCDs	Three or more NCDs
	***N*** = 13,798	***N*** = 5197	***N*** = 5568	***N*** = 2006	***N*** = 1027
**Health care utilisation**					
Individual reported outpatient care (%)	20.80 (20.08–21.55)	13.31 (12.26–14.39)	19.96 (18.77–21.14)	32.12 (29.90–34.34)	44.83 (41.64–48.02)
Frequency of outpatient care	0.39 (0.37–0.42)	0.21 (0.19–0.24)	0.35 (0.32–0.38)	0.68 (0.61–0.74)	1.08 (0.96–1.21)
Individual reported inpatient care (%)	4.35 (4.01–4.69)	1.83 (1.46–2.15)	3.85 (3.32–4.38)	7.82 (6.63–9.00)	14.34 (11.93–16.75)
Frequency of inpatient care	0.60 (0.05–0.06)	0.02 (0.02–0.03)	0.05 (0.04–0.05)	0.11 (0.09–0.13)	0.24 (0.18–0.30)
**Out-of-pocket expenditure (OOPE)**					
Any OOPE (%)	15.70 (15.02–16.38)	10.76 (9.79–11.72)	15.43 (14.36–16.50)	22.62 (20.57–24.67)	30.94 (27.89–33.99)
OOPE for outpatient care^a^	$29.0 (19.7–38.4)	$17.2 (13.9–20.5)	$23.5 (16.6–30.5)	$46.7 (8.5–85.0)	$37.5 (16.5–58.4)
OOPE for inpatient care^b^	$877 (632.9–1121.3)	$774.6 (500.9–1048.2)	$655.6 (406.3–904.9)	$607.7 (324.5–890.9)	$1594.3 (670.4–2518.1)
Average four-weekly OOPE ^c^	$38.7 (29.7–47.6)	$23.1 (18.2–28.0)	$29.8 (22.7–36.8)	$51.1 (17.4–84.8)	$69.3 (40.3–98.4)

*NCDs* non-communicable diseases, *OOPE* out-of-pocket expenditure

^a^In the last 4 weeks. Respondents who reported outpatient care = 2894

^b^In the last 12 months. Respondents who reported inpatient care = 630

^c^Detailed explanation of the calculation of four-weekly OOPE is available in the method section. Respondents who reported out/inpatient care(s) = 3243

Values are unweighted counts and weighted percentages

Bootstrapping with 500 times replications was performed to estimate the standard error. Numbers in bracket represent the 95% confidence interval

**Fig. 1:**
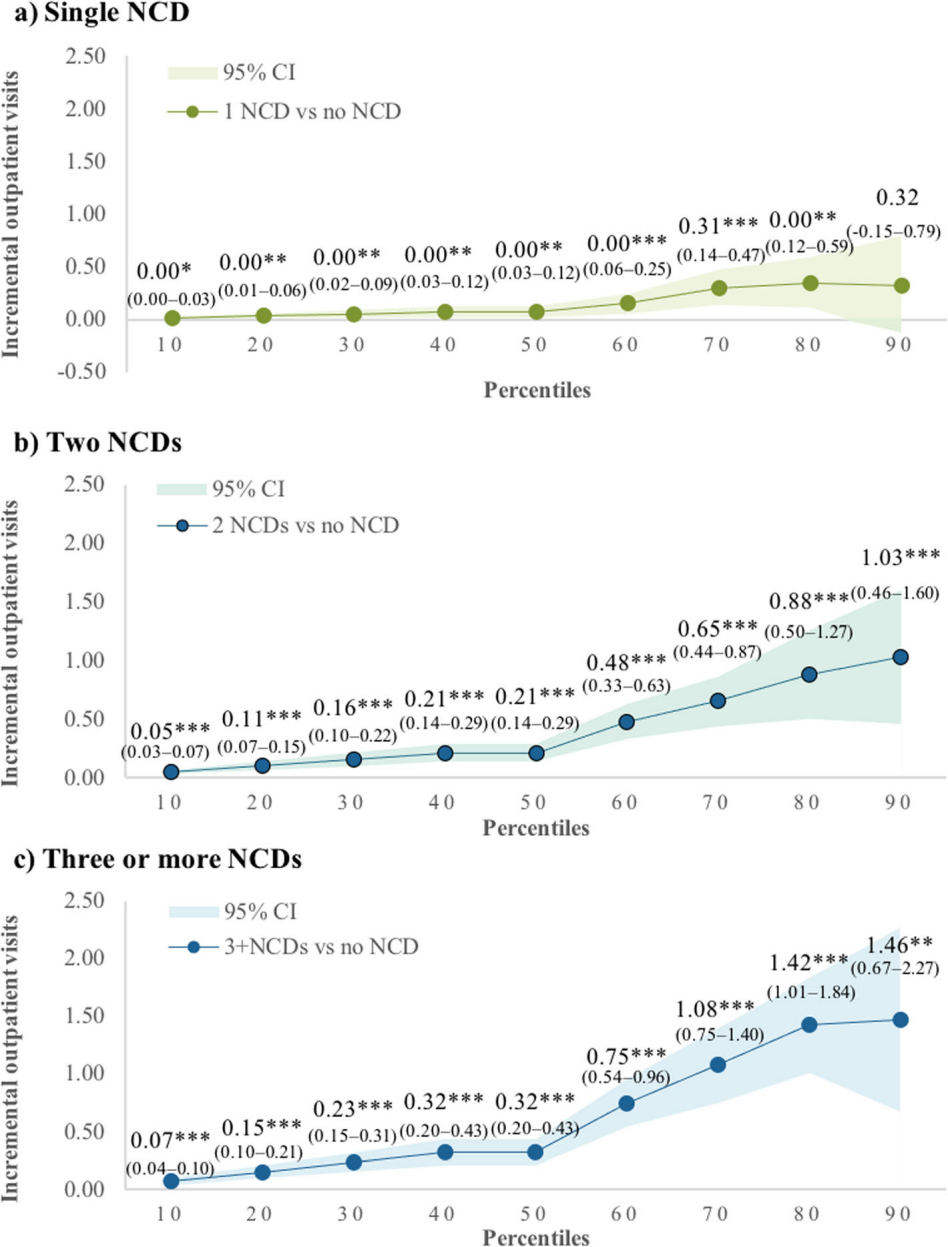
Marginal effects of the number of NCDs on outpatient visits by percentiles of outpatient visits*.* Notes: CI — confidence interval; NCDs — non-communicable diseases*.* *p-value < 0.05 **p-value < 0.01 ***p-value < 0.001*.* Respondents who reported at least one outpatient visit = 2894*.* Bootstrapping with 500 times replications was performed to estimate the standard error. Shaded area in the graph represents the confidence interval

Figure 1 and Additional file 1: Appendix 4 present the results of logistic regression and quantile regression analysis for outpatient care. Our findings show that compared with those without any NCD, the odds of having outpatient care were 2.73 times (95% CI 2.38–3.14) and 4.51 times (95% CI 3.81–5.33) higher among respondents with two or three or more NCDs, respectively (Additional file 1: Appendix 4). Figure 1 shows that compared with respondents having no NCDs, having more NCDs was associated with an increased number of outpatient care across the whole distribution of outpatient care. The marginal effects were small at the bottom percentiles, but more pronounced at the higher percentiles of outpatient care. For example, at the 10th percentile, those having two NCDs had an increase of 0.05 (95% CI 0.03–0.07) outpatients visit compared with those without NCD (Fig. 1b). The marginal effect increased at the 90th percentile, where having two NCDs was associated with an increase of 1.03 (95% CI 0.46–1.60) outpatient care compared with those without NCD. The differences between percentiles suggest the differential effects of the number of NCDs on outpatient care across the distribution of outpatient care utilisation. Furthermore, the higher the number of NCDs, the greater the difference between lower and upper percentiles. The marginal effects of having three or more NCDs on outpatient care at the 90th percentile was 1.46 (95% CI 0.67–2.27) visit compared with 0.07 (95% CI 0.04–0.10) visit at the 10th percentile of outpatient care (Fig. 1c).

**Fig. 2:**
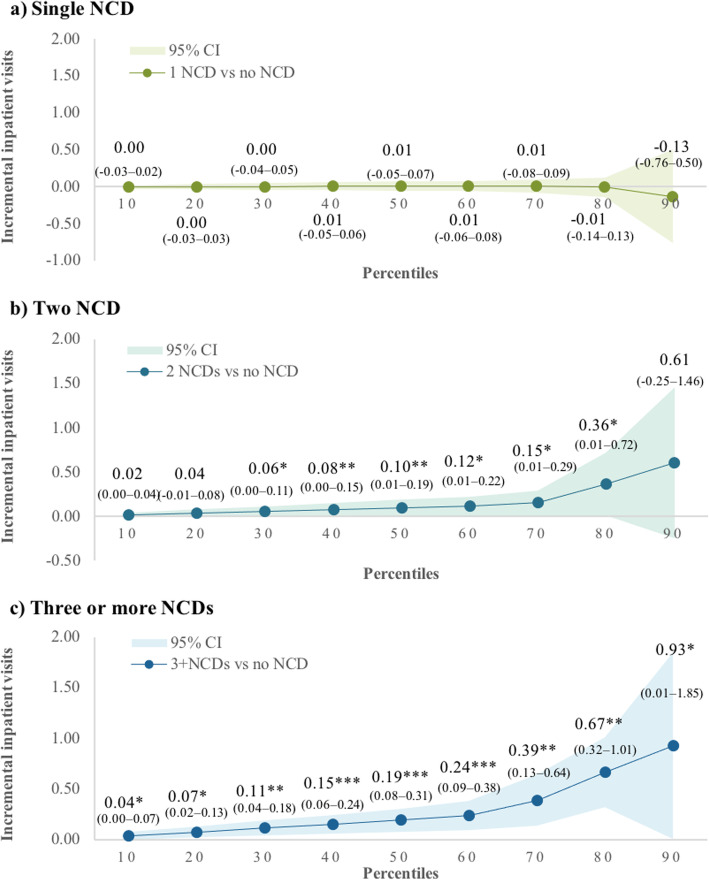
Marginal effects of the number of NCDs on inpatient visits by percentiles of inpatient visits. Notes: CI — confidence interval; NCDs — non-communicable diseases. *p-value < 0.05 **p-value < 0.01 ***p-value < 0.001. Respondents who reported at least one inpatient visit = 630. Bootstrapping with 500 times replications was performed to estimate the standard error. Shaded area in the graph represents the confidence interval

Similarly, having multimorbidity also increased the odds of experiencing inpatient care. For example, the odds of having inpatient care were 3.60 times (95% CI 2.69–4.83) higher in those with two NCDs. The odds almost doubled to 6.67 times (95% CI 4.92–9.05) for respondents with three or more NCDs (Additional file 1: Appendix 5). Figure 2 indicates that having two NCDs or three or more NCDs also increased the number of inpatient care in almost all distribution of inpatient care utilisation (Fig. 2b and c). The marginal effect of having three NCDs or more on inpatient care at the 90th percentile was 0.93 (95% CI 0.01–1.85) visit compared with 0.04 (95% CI 0.00–0.07) visits at the 10th percentile of inpatient care utilisation.

**Fig. 3:**
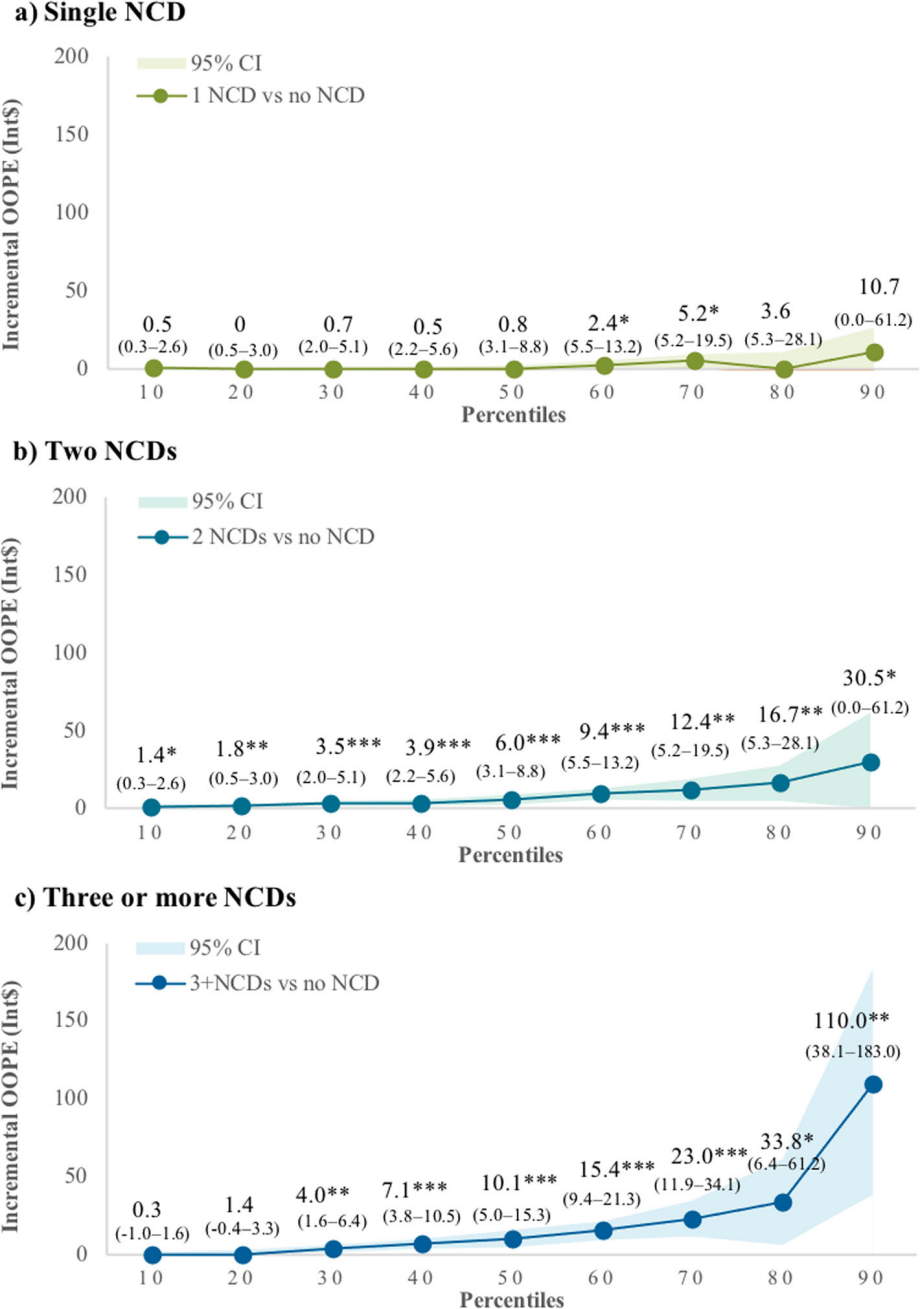
Marginal effects of the number of NCDs on four-weekly OOPE by percentiles of OOPE. Notes: CI — confidence interval; NCDs — non-communicable diseases; OOPE — out-of-pocket expenditure. *p-value < 0.05 **p-value < 0.01 ***p-value < 0.001. Respondents who had OOPE (OOPE ≥ Int$1) = 2097. Bootstrapping with 500 times replications was performed to estimate the standard error. Shaded area in the graph represents the confidence interval

The presence of NCDs was associated with greater OOPE (Fig. 3 and Additional file 1: Appendix 6). Individuals diagnosed with two or three or more NCDs had 2.39 times (95% CI 2.04–2.80) and 3.55 times (95% 2.96–4.27) higher odds of paying out-of-pocket, respectively (Additional file 1: Appendix 6). The result of quantile regression analysis among those who had OOPE (OOPE ≥ Int$1) suggests that an increased number of NCDs was significantly associated with greater OOPE at higher percentiles compared to lower percentiles of OOP spending (Fig. 3). Respondents with two NCDs (Fig. 3b) had an increased four-weekly OOPE of Int$1.4 (95% CI $0.3–2.6), Int$6.0 (95% CI $3.1–8.8), Int$30.5 (95% CI $0.0–61.1), at the 10th, 50th, and 90th percentiles, respectively, compared with those without any NCDs. The incremental OOPE of having three NCDs or more (Fig. 3c) was significantly higher compared with those without any NCDs at the 30th percentile and higher, for example, Int$4.0 (95% CI $1.6–6.4), Int$10.1 (95% CI $5.0–15.3), and Int$110.0 (95% CI $38.1–183.0) at the 30th, 50th, and 90th percentiles, respectively. Conversely, the effect of a single NCD on OOPE was not significant across the percentiles, except for the 60th and 70th percentile (Fig. 3a). We also present the predictive OOPE by number of NCDs in Additional file 1: Appendix 7. At the 90th percentile, those who had three or more NCDs were predicted to have significantly higher OOPE of Int$204.0 (95% CI 131.0–277.0) per 4 weeks compared with those with one NCD with Int$104.1 (95% CI 84.0–124.0). Despite having health insurance reduce the odds of paying out-of-pocket (aOR 0.84, 95% CI 0.76–0.94), it did not statistically significantly reduce OOPE in all percentiles of OOP spending (Additional file 1: Appendix 6).

We present the mean of outpatients, inpatient, and OOPE by population quintile in Additional file 1: Appendix 8. The average number of outpatient care ranges between 0.07 (95% CI 0.07–0.08) at the 10th percentile and 1.20 (95% CI 1.14–1.25) at the 90th percentile of outpatient care utilisation (Additional file 1: Appendix 8). We further estimated the effects of the number of NCDs on outcomes by quantile in Table 3. An increased number of NCDs was significantly associated with a higher number of outpatient care across every percentile group. The marginal effects of NCDs were found to be larger among the higher percentiles of outpatient care utilisation than those in the lower percentiles (for 10th percentile, coefficients 0.02, 95% CI 0.01–0.03; for 90th percentile, coefficients 0.13, 95% CI 0.08–0.17).

**Table 3:** The incremental outpatient care, inpatient care, and average four-weekly OOPE for an additional NCD reported, by population quintile

Variables	Overall		Quantile regression									
			10th percentile		25th percentile		50th percentile		75th percentile		90th percentile	
	Coef^a^	95% CI	Coef	95% CI	Coef	95% CI	Coef	95% CI	Coef	95% CI	Coef	95% CI
**Frequency of outpatient care**												
Number of NCDs	0.11^***^	(0.07–0.14)	0.02^***^	(0.01–0.03)	0.05^***^	(0.03–0.06)	0.09^***^	(0.07–0.12)	0.16^***^	(0.12–0.19)	0.13^***^	(0.08–0.17)
**Frequency of inpatient care**												
Number of NCDs	0.09^**^	(0.02–0.16)	0.01^*^	(0.00–0.02)	0.02^**^	(0.01–0.04)	0.04^***^	(0.02–0.07)	0.10^***^	(0.06–0.15)	0.17^**^	(0.05–0.30)
**Average four-weekly out-of-pocket expenditure (OOPE)**												
Number of NCDs	27.0^**^	(11.4–42.7)	0.3	(−0.1–0.7)	1.0^***^	(0.5–1.5)	3.1^***^	(2.0–4.2)	9.5^***^	(5.6–13.2)	31.0^***^	(15.9–46.2)

*CI* confidence interval, *NCDs* non-communicable diseases, *OOPE* out-of-pocket expenditure

*p-value < 0.05 **p-value < 0.01 ***p-value < 0.001

Bootstrapping with 500 times replications was performed to estimate the standard error

^a^Estimated using Poisson regression models (outpatient and inpatient care) and ordinary least square (OOPE)

For inpatient care, the average number of inpatient care ranges from 0.02 (95% CI 0.02–0.03) at the 10th percentile to 0.64 (95% CI 0.56–0.75) at the 90th percentile of inpatient care utilisation (Additional file 1: Appendix 8). Having more NCDs was associated with an increase in the number of inpatient care. The magnitude of the effect of the number of NCDs was more pronounced for the upper percentiles (coefficients 0.17, 95% CI 0.05–0.30 for 90th percentile), compared with bottom percentiles (coefficients 0.01, 95% CI 0.00–0.02 for 10th percentile) of inpatient care utilisation (Table 3).

For OOPE, the average amount of OOPE ranges from Int$7.6 at the 10th percentile (95% CI $6.5–8.7) to Int$101.0 at the 90th percentile (95% $88.3–115.0) of OOP spending (Additional file 1: Appendix 8). The magnitude of the effect of the number of NCDs was greater at upper percentiles compared with lower percentiles of OOPE spending (Table 3, coefficients 1.0, 95% CI 0.5–1.5 for the 25th percentile; coefficients 31.0, 95% CI 15.9–46.2 for the 90th percentile).

## Discussion

### Principal findings

Our findings reveal that one in five of the study participants, over 40 years of age, had at least two NCDs and that the prevalence of multimorbidity varied across sociodemographic characteristics. Increasing age, female gender, urban residency, and high per-capita expenditure were some of the significant factors of multimorbidity. Consistent with earlier studies [19, 28–31], our findings suggest that the presence of multimorbidity was significantly associated with higher inpatient/outpatient care, as well as higher individual OOPE. Notably, our results from quantile regression analysis revealed a more detailed measure of the association between multimorbidity and OOPE that had not been revealed in earlier published studies that have used OLS or GLM in the analysis. Our results indicated that the effect of multimorbidity is more pronounced among those at a higher percentile of the health utilisation/OOPE distribution. Multimorbidity has a significant association with cost at various points in the outcome distribution, even for those with low utilisation/OOPE. Our findings corroborate with an earlier study in Indonesia that showed that NCDs are associated with higher OOPE [16]. However, this earlier study had only focused on the burden of a single disease, which is increasingly common in Indonesia. Our quantile regression analysis provides a more detailed and comprehensive assessment of the association between multimorbidity, health care use, and OOPE than any previous studies in the literature.

### Strength and limitation

To the best of our knowledge, this is the first study in Indonesia to estimate the burden of OOPE associated with multimorbidity using nationally representative data. Besides estimating the overall attributable OOPE to multimorbidity, quantile regression were applied to estimate the impact of multimorbidity on different population income groups across different parts of the distribution of health care utilisation and four-weekly OOPE.

This study, however, has several limitations. Firstly, the use of self-reported measures of chronic conditions may lead to an underestimation of the prevalence of multimorbidity, particularly for older adults and those from lower socioeconomic and educational backgrounds who may be more likely to have limited access to health care. The 2018 Basic Health Research (Riskesdas) revealed that only 2.0% of the sample self-reported having diabetes, compared to 10.9% having diabetes based on blood sample measurements [32]. Additionally, the self-reported OOPE in the IFLS-5 might be prone to recall bias, which would lead to the imprecision of estimated OOPE. Moreover, the survey estimated aggregate expenditure for both inpatient and outpatient care, which may provide a higher estimate of health spending [33]. Secondly, we divided the annual out-of-pocket for inpatient care by 13 to calculate the average four-weekly OOPE. This assumption was used because the survey collected data on outpatient care in the previous four-week range, while the history of inpatient care was collected in a one-year range. Thirdly, the effect of multimorbidity on healthcare utilisation and OOPE was examined by simply counting the number of NCDs without accounting for the different clusters and severity of NCDs. Future studies are warranted to take into account the severity of NCDs by applying appropriate weighting for each NCD [34]. Finally, the original IFLS-5 sampling frame did not include the Eastern regions of Indonesia, which are considered underdeveloped. According to 2018 Riskesdas, the prevalence of self-reported NCDs, including asthma, stroke, cardiovascular diseases, diabetes, and high blood pressure, in the Eastern region of Indonesia was lower than the national average [32]. The multimorbidity assessment of the remaining Indonesian regions, as well as prospective study designs, should be considered for future studies.

### Policy and clinical implications

Our findings provide timely evidence on the economic burden of multimorbidity in Indonesia. A study conducted by Husnayain et al. found that 43% of patients who utilised secondary health care between 2015 and 2016 in Indonesia had multimorbidity [35]. Thus, our finding highlights the urgent need for policy interventions to reduce the financial burden for patients with multimorbidity in Indonesia. Despite the introduction of a national health insurance program (JKN) in 2014, several studies reported that expenditure on medicines still occurs as the major contributor to OOPE [36, 37]. One of the main reasons is the unavailability of JKN-covered drugs in the health facilities, forcing JKN participants to purchase drugs not covered by JKN [36, 38]. OOPE for medicines is particularly important to address for patients with multimorbidity, where the high costs of long-term medications would negatively impact household’s economies [14, 37].

The greater prevalence of multimorbidity among populations with higher socioeconomic status underscores the importance of targeted interventions for patients with higher risks of developing NCDs and multimorbidity in Indonesia. Evidence suggests that people living with NCDs are mostly not aware of their condition. Therefore, the health care system and financing need to be re-oriented to promotive and preventive efforts [39]. A critical component to achieve greater health promotive and preventive efforts is the strengthening of the primary healthcare (PHC) system [40, 41]. PHC plays an integral role in promoting healthy lifestyle behaviours, early detection of NCDs and the risk factors, following-up patients with NCDs [41, 42]. Investing in PHC would be more effective and less costly to prevent and manage multimorbidity by having multidisciplinary teams led by general practitioners. Yet, in many poor settings, the capacity and responsiveness of PHC to cope with the imminent burden of NCD epidemics are still weak [43]. While Indonesia has adopted WHO PEN (Package for essential noncommunicable disease interventions for primary healthcare in low-resource settings) to improve access to NCD screening and management, the evidence of its implementation is still lacking [44].

In contrast to findings in HICs, our study found that individuals in the higher per capita expenditure quantile higher educational attainment (a proxy for higher socioeconomic status) were more likely to report multimorbidity. Earlier studies in LMICs have also shown a higher prevalence of NCDs in higher socioeconomic groups [45] as these countries undergo epidemiological transition [46–49]. This may be partly explained by higher-income groups, who have better access to healthcare services and better health literacy, receiving diagnoses of their conditions to a greater extent than lower-income groups. The presence of chronic conditions may be more likely to be reported when a diagnosis has been received.

In the case of Indonesia, this socio-economically better off population subgroup would be more likely to access private PHC centers, which may operate differently in terms of health promotion programs compared to public PHC [50]. Private PHC, in general, is not designed to provide robust health promotion and disease prevention programs, as well as active follow up of patients with chronic illnesses. Therefore, there is a need to strengthen collaboration between the public and private healthcare sectors, especially important in the case of Indonesia where the private sector accounts for 60% of health facilities [51].

## Conclusion

Multimorbidity is positively correlated with health service utilisation and has a significant effect on OOPE, especially among those in the upper tail of the outcome distribution. These results underscored the importance of more detailed and clear evidence on the economic consequences of multimorbidity to inform the cost-effectiveness of policies and prevention strategies.

## Supplementary Information

**Additional file 1: Appendix 1**. Sample flowchart. **Appendix 2.** List of variables for 2014 IFLS-5 analysis. **Appendix 3**. Sample characteristics of respondents with multimorbidity. **Appendix 4.** The incremental outpatient visits by population quantile. **Appendix 5.** The incremental inpatient visits by population quantile. **Appendix 6.** The incremental out-of-pocket expenditure by population quantile. **Appendix 7.** Predictive four-weekly OOPE by number of NCDs. **Appendix 8.** Average number of outpatient visit, inpatient visits, and four-weekly OOPE by population quantile.

## Data Availability

The datasets supporting the conclusions of this article are available after registration in https://www.rand.org/well-being/social-and-behavioral-policy/data/FLS/IFLS/access.html.

## Abbreviations

IFLS: Indonesian Family Life Survey
JKN: National Health Insurance
LMICs: Low-income and middle-income countries
NCDs: Non-communicable diseases
OOPE: Out-of-pocket expenditure
PHC: Primary health care
