# Supplementary material for: Effect of multimorbidity on utilisation and out-of-pocket expenditure in Indonesia: quantile regression analysis
Source: BMC Health Serv Res. 2021 May 5;21:427. doi: 10.1186/s12913-021-06446-9 (PMC8097787; doi:10.1186/s12913-021-06446-9)
Supplement: Supplementary file 1 — Additional file 1: Appendix 1. Sample flowchart. Appendix 2. List of variables for 2014 IFLS-5 analysis. Appendix 3. Sample characteristics of respondents with multimorbidity. Appendix 4. The incremental outpatient visits by population quantile. Appendix 5. The incremental inpatient visits by population quantile. Appendix 6. The incremental out-of-pocket expenditure by population quantile. Appendix 7. Predictive four-weekly OOPE by number of NCDs. Appendix 8. Average number of outpatient visit, inpatient visits, and four-weekly OOPE by population quantile. [file 12913_2021_6446_MOESM1_ESM.docx]

# Effect of multimorbidity on utilisation and out-of-pocket expenditure in Indonesia: quantile regression analysis

Kanya Anindya^1^*, Nawi Ng^2^, Rifat Atun^3^, Tiara Marthias^1,4^, Yang Zhao^5,6^, Barbara McPake^1^, Alexander van Heusden^1^, Tianxin Pan^7^, John Tayu Lee^1,8^

^1^ The Nossal Institute for Global Health, Melbourne School of Population and Global Health, The University of Melbourne, Melbourne, VIC, Australia

^2^ Department of Public Health and Community Medicine, Institute of Medicine, Sahlgrenska Academy, University of Gothenburg, Gothenburg, Sweden

^3^ Department of Global Health and Population, Harvard T.H. Chan School of Public Health, Harvard University, Boston, MA, USA

^4^ Department of Public Health, Faculty of Medicine, Public Health and Nursing, Universitas Gadjah Mada, Yogyakarta, Indonesia

^5^ The George Institute for Global Health at Peking University Health Science Center, Beijing, China

^6^ WHO Collaborating Centre on Implementation Research for Prevention and Control of Noncommunicable Diseases, Melbourne, VIC, Australia

^7^ Centre for Health Policy, School of Population and Global Health, The University of Melbourne, Melbourne, Australia

^8^ Department of Primary Care and Public Health, School of Public Health, Imperial College London, London, UK

* Corresponding author

E-mail address: kanindya@student.unimelb.edu.au (KA)

Telephone number: +62 878 9966 9201

**Supplementary information**

**Additional file 1. Appendices**

**Appendix 1**. Sample flowchart

**Appendix 2.** List of variables for 2014 IFLS-5 analysis

**Appendix 3**. Sample characteristics of respondents with NCD multimorbidity

**Appendix 4.** The incremental outpatient visits by population quantile

**Appendix 5.** The incremental inpatient visits by population quantile

**Appendix 6.** The incremental out-of-pocket expenditure by population quantile

**Appendix 7.** Predictive OOPE by number of NCDs

**Appendix 8.** Average number of outpatient visit, inpatient visits, and total amount of OOPE by population quantile

**Appendix 1. Sample flowchart**

**Appendix 2. List of variables for 2014 IFLS-5 analysis**

| **Variables** | **Type** | **Measurement** | **Source of measurement** |
| --- | --- | --- | --- |
| **Dependent variables:** | | | |
| 1. Health service use | | | |
| Outpatient care | Binary  Numerical | 0. No  1. Yes  Number of days | RJ00: In the last 4 weeks have you visited a public hospital-*puskesmas*-private hospital-clinic-health worker or doctor’s practice or been visited by a health worker or doctor?  RJ02: How many times did you visit / been visited by [...] during the last 4 weeks? |
| Inpatient care | Binary  Numerical | 0. No  1. Yes  Number of days | RN00: During the past 12 months have you ever received patient care at a hospital-*puskesmas*-clinic-or  other?  RN02: How many times have you received inpatient care at […] during the past 12  months? |
| 1. Financial burden | | | |
| Annual Total OOPE | Numerical | International Dollars | Annual total OOPE for outpatient and inpatient visits |
| **Main independent variable** | | | |
| Number of NCDs | Numerical  Categorical ordinal | Total number of chronic conditions related to NCDs   1. No 2. 1 NCD 3. 2 NCDs 4. 3+ NCDs | Book IIIB:  CD06a – CD06r: Have a  doctor/paramedic/nurse/ midwife ever told you that you had [list of chronic diseases] |
| Multimorbidity | Binary | 1. No 2. Yes (had 2 or more chronic conditions related to NCDs) | Book IIIB:  CD06a – CD06r: Have a  doctor/paramedic/nurse/ midwife ever told you that you had [list of chronic diseases] |
| List of chronic diseases included in the main analysis:  Hypertension, diabetes, asthma, heart attack/coronary heart diseases, liver disease, stroke, cancer, arthritis/rheumatism, hypercholesterolemia, mental illness, prostate diseases, kidney diseases (excluding malignancy), digestive diseases, and memory-related diseases. | | | |
| **Covariates** | | | |
| Age (in years) | Categorical ordinal | 1. 40-49 years 2. 50-59 years 3. 60-69 years 4. 70-79 years 5. 80+ | Book IIIA:  Age: How old are you? |
| Sex | Binary | 1. Male 2. Female | Book IIIA:  Sex: (identified by interviewers) |
| Marital status | Binary | 1. Unmarried/Divorce 2. Married or living together | Book IIIA  HR00b: Are you currently married? |
| Education | Categorical ordinal | 1. None 2. Elementary school 3. Junior high school 4. High school 5. Tertiary | Book IIIA:  DL06: What is the highest education level  attended?  DL07: What is the highest grade completed at school. |
| Residency | Binary | 1. Rural 2. Urban | Book T-2:  SC06: (identified by interviewers) |
| Region | Categorical nominal | 1. Java-Bali 2. Sumatra 3. Nusa Tenggara 4. Kalimantan 5. Sulawesi | Book T-2:  SC01: province (identified by interviewers) |
| Health insurance status | Binary | 1. Uninsured (Not covered by any insurance) 2. Insured | Book IIIB:  AK01: Are you the policy holder/primary beneficiary of health benefits-health insurance? |
| Per capita expenditure | Categorical ordinal | 1. Q1 (lowest) 2. Q2 3. Q3 4. Q4 5. Q5 (highest) | Book KS |

**Appendix 3. Sample characteristics of respondents with NCD multimorbidity**

| **Characteristics** | **Total** | | **Mean number of NCDs** | | **Percentages with multimorbidity*** | |
| --- | --- | --- | --- | --- | --- | --- |
|  | n | % | Mean | 95% CI | n | % (95% CI) |
| **Overall** | 13,798 | 100.0 | 0.92 | (0.90–0.94) | 3033 | 20.84 (20.12–21.57) |
| **Age (year)** |  |  |  |  |  |  |
| 40 – 49 years | 5872 | 44.63 | 0.71 | (0.69–0.74) | 872 | 14.70 (13.75–15.70) |
| 50 – 59 years | 3999 | 29.02 | 1.00 | (0.96–1.03) | 969 | 23.28 (21.90–24.72) |
| 60 – 69 years | 2210 | 15.46 | 1.19 | (1.14–1.23) | 682 | 29.06 (27.07–31.11) |
| 70+ years | 1717 | 10.89 | 1.19 | (1.14–1.24) | 510 | 27.77 (25.53–30.09) |
| **Gender** |  |  |  |  |  |  |
| Male | 6627 | 49.00 | 0.81 | (0.79–0.84) | 1196 | 17.18 (16.23–18.17) |
| Female | 7171 | 51.00 | 1.03 | (1.00–1.05) | 1837 | 24.34 (23.29–25.42) |
| **Marital status** |  |  |  |  |  |  |
| Not currently married | 2928 | 19.68 | 1.07 | (1.03–1.10) | 780 | 25.22 (23.57–26.95) |
| Currently married | 10870 | 80.32 | 0.89 | (0.98–0.91) | 2253 | 19.76 (18.97–20.57) |
| **Education status** |  |  |  |  |  |  |
| No education | 5359 | 39.36 | 0.92 | (0.89–0.95) | 1136 | 19.60 (18.49–20.77) |
| Primary | 3237 | 24.79 | 0.90 | (0.86–0.93) | 703 | 20.26 (18.83–21.76) |
| Junior high school | 1570 | 11.13 | 0.91 | (0.86–0.97) | 339 | 21.12 (19.05–23.34) |
| Senior high school | 2587 | 17.67 | 0.89 | (0.85–0.93) | 552 | 20.82 (19.20–22.54) |
| Tertiary | 1045 | 7.050 | 1.11 | (1.03–1.18) | 303 | 29.30 (26.40–32.37) |
| **Residency** |  |  |  |  |  |  |
| Rural | 5850 | 49.07 | 0.84 | (0.82–0.87) | 1078 | 17.48 (16.46–18.55) |
| Urban | 7948 | 50.93 | 1.00 | (0.97–1.02) | 1955 | 24.06 (23.08–25.08) |
| **Region** |  |  |  |  |  |  |
| Java-Bali | 8694 | 75.71 | 0.92 | (0.90–0.94) | 1909 | 20.52 (19.65–21.41) |
| Sumatra | 2920 | 16.1 | 0.96 | (0.92–1.00) | 706 | 23.32 (21.76–24.87) |
| Nusa Tenggara | 863 | 2.60 | 0.64 | (0.59–0.69) | 104 | 10.85 (9.00–13.02) |
| Kalimantan | 602 | 2.56 | 1.04 | (0.95–1.11) | 165 | 25.70 (22.38–29.31) |
| Sulawesi | 719 | 3.03 | 0.89 | (0.82–0.96) | 149 | 20.36 (17.46–23.37) |
| **PCE, quartile** |  |  |  |  |  |  |
| Q1 (the lowest) | 2761 | 21.36 | 0.79 | (0.75–0.82) | 426 | 14.79 (13.44–16.26) |
| Q2 | 2761 | 21.04 | 0.84 | (0.80–0.87) | 525 | 18.36 (16.86–19.96) |
| Q3 | 2766 | 19.78 | 0.92 | (0.88–0.96) | 599 | 21.02 (19.43–22.71) |
| Q4 | 2759 | 19.46 | 0.97 | (0.92–1.01) | 656 | 22.55 (20.92–24.27) |
| Q5 (the highest) | 2751 | 18.37 | 1.14 | (1.10–1.18) | 827 | 28.66 (26.88–30.50) |
| **Had any health insurance** | |  |  |  |  |  |
| No | 6925 | 52.1 | 0.86 | (0.83–0.88) | 1314 | 18.05 (17.10–19.04) |
| Yes | 6873 | 47.9 | 0.99 | (0.97–1.02) | 1719 | 23.86 (22.80–24.96) |
| NCDs — noncommunicable diseases–PCE — per capita expenditure  *We defined multimorbidity if the respondents reported that they had 2 or more chronic conditions related to NCDs. Chronic diseases in IFLS5 included hypertension, diabetes mellitus, asthma, chronic heart diseases, mental health issue, stroke, liver diseases, cancer/malignancies, liver, arthritis, high cholesterol, prostate illness kidney diseases, digestive system diseases. Values are unweighted counts and weighted percentages | | | | | | |

**Appendix 4. The incremental outpatient visits by population quintile**

| **Variables** | **Logistic regression (n=13,798)**^*^ | | **Quantile Regression**  **(n=2,894)**^†^ | | | | | | | | | |
| --- | --- | --- | --- | --- | --- | --- | --- | --- | --- | --- | --- | --- |
|  |  |  | 10^th^ percentile | | 25^th^ percentile | | 50^th^ percentile | | 75^th^ percentile | | 90^th^ percentile | |
|  | OR | (95% CI) | Coef. | (95% CI) | Coef. | (95% CI) | Coef. | (95% CI) | Coef. | (95% CI) | Coef. | (95% CI) |
| Number of NCDs  (ref. no NCD) |  |  |  |  |  |  |  |  |  |  |  |  |
| One | **1.52** | **(1.35–1.70)** | **0.02** | **(0.00–0.03)** | **0.04** | **(0.01–0.07)** | **0.07** | **(0.03–0.12)** | **0.19** | **(0.08–0.29)** | **0.11** | **(-0.05–0.27)** |
| Two | **2.73** | **(2.38–3.14)** | **0.05** | **(0.03–0.07)** | **0.11** | **(0.07–0.15)** | **0.21** | **(0.14–0.28)** | **0.36** | **(0.23–0.48)** | **0.32** | **(0.15–0.48)** |
| Three or more | **4.51** | **(3.81–5.33)** | **0.07** | **(0.04–0.09)** | **0.16** | **(0.10–0.21)** | **0.32** | **(0.22–0.43)** | **0.54** | **(0.40–0.69)** | **0.42** | **(0.22–0.62)** |
| Age (year)  (ref. 40 – 49 years) |  |  |  |  |  |  |  |  |  |  |  |  |
| 50 – 59 years | 1.05 | (0.93–1.18) | 0.00 | (-0.01–0.02) | 0.00 | (-0.03–0.03) | 0.01 | (-0.05–0.06) | -0.00 | (-0.10–0.10) | -0.08 | (-0.21–0.05) |
| 60 – 69 years | 1.14 | (0.99–1.32) | 0.00 | (-0.02–0.02) | 0.01 | (-0.03–0.05) | 0.00 | (-0.06–0.07) | 0.02 | (-0.12–0.15) | -0.00 | (-0.17–0.16) |
| 70+ years | **1.21** | **(1.02–1.43)** | -0.00 | (-0.03–0.02) | -0.01 | (-0.05–0.04) | -0.01 | (-0.10–0.07) | 0.03 | (-0.11–0.17) | 0.01 | (-0.19–0.21) |
| Gender  (ref. Female) |  |  |  |  |  |  |  |  |  |  |  |  |
| Male | **1.47** | **(1.33–1.62)** | -0.00 | (-0.01–0.01) | -0.00 | (-0.03–0.02) | -0.00 | (-0.05–0.04) | -0.02 | (-0.10–0.07) | 0.03 | (-0.09–0.15) |
| Marital status |  |  |  |  |  |  |  |  |  |  |  |  |
| Currently married | 1.09 | (0.96–1.23) | -0.01 | (-0.02–0.01) | -0.01 | (-0.05–0.02) | -0.02 | (-0.08–0.05) | 0.03 | (-0.06–0.13) | 0.08 | (-0.06–0.22) |
| Education status |  |  |  |  |  |  |  |  |  |  |  |  |
| Primary | 0.97 | (0.86–1.10) | -0.01 | (-0.03–0.00) | **-0.04** | **(-0.07–-0.00)** | **-0.07** | **(-0.13–-0.01)** | -0.10 | (-0.22–0.01) | -0.10 | (-0.24–0.04) |
| Junior high school | 1.01 | (0.85–1.19) | -0.00 | (-0.03–0.02) | -0.01 | (-0.06–0.03) | -0.04 | (-0.12–0.05) | -0.04 | (-0.19–0.10) | -0.11 | (-0.26–0.05) |
| Senior high school | 0.89 | (0.76–1.03) | **-0.03** | **(-0.05–-0.01)** | **-0.07** | **(-0.11–-0.03)** | **-0.14** | **(-0.21–-0.07)** | **-0.25** | **(-0.38–-0.12)** | **-0.23** | **(-0.42–-0.04)** |
| Tertiary | 0.91 | (0.75–1.11) | **-0.04** | **(-0.06–-0.02)** | **-0.09** | **(-0.14–-0.04)** | **-0.17** | **(-0.26–-0.08)** | **-0.32** | **(-0.46–-0.18)** | **-0.46** | **(-0.71–-0.21)** |
| Residency  (ref. Rural) |  |  |  |  |  |  |  |  |  |  |  |  |
| Urban | 0.98 | (0.88–1.08) | 0.00 | (-0.01–0.01) | 0.00 | (-0.03–0.03) | 0.01 | (-0.04–0.06) | 0.00 | (-0.09–0.09) | 0.01 | (-0.10–0.13) |
| Region |  |  |  |  |  |  |  |  |  |  |  |  |
| Sumatra | **0.80** | **(0.72–0.90)** | 0.00 | (-0.01–0.02) | 0.01 | (-0.03–0.04) | 0.01 | (-0.05–0.07) | 0.05 | (-0.07–0.16) | 0.06 | (-0.08–0.21) |
| Nusa Tenggara | **0.92** | **(0.76–1.12)** | -0.02 | (-0.04–0.00) | **-0.05** | **(-0.09–-0.00)** | **-0.09** | **(-0.17–-0.01)** | -0.11 | (-0.30–0.09) | -0.09 | (-0.29–0.12) |
| Kalimantan | **0.77** | **(0.62–0.95)** | -0.00 | (-0.03–0.03) | -0.01 | (-0.08–0.06) | -0.02 | (-0.14–0.10) | -0.06 | (-0.24–0.12) | -0.06 | (-0.36–0.23) |
| Sulawesi | **0.62** | **(0.50–0.76)** | -0.02 | (-0.05–0.01) | **-0.05** | **(-0.11–-0.00)** | **-0.10** | **(-0.20–-0.01)** | -0.12 | (-0.32–0.09) | -0.16 | (-0.36–0.05) |
| PCE^‡^ (ref. Q1) |  |  |  |  |  |  |  |  |  |  |  |  |
| Q2 | **1.30** | **(1.11–1.52)** | **0.02** | **(0.00–0.04)** | **0.05** | **(0.01–0.09)** | **0.10** | **(0.02–0.18)** | **0.21** | **(0.07–0.36)** | **0.23** | **(0.06–0.40)** |
| Q3 | **1.50** | **(1.28–1.76)** | -0.00 | (-0.02–0.02) | -0.00 | (-0.04–0.04) | -0.01 | (-0.08–0.06) | 0.03 | (-0.10–0.17) | 0.10 | (-0.09–0.28) |
| Q4 | **1.80** | **(1.54–2.11)** | 0.01 | (-0.01–0.03) | 0.02 | (-0.01–0.06) | 0.05 | (-0.03–0.12) | 0.12 | (-0.01–0.25) | 0.16 | (-0.01–0.34) |
| Q5 | **1.74** | **(1.48–2.05)** | **0.02** | **(0.00–0.04)** | **0.05** | **(0.01–0.10)** | **0.10** | **(0.02–0.18)** | **0.23** | **(0.10–0.35)** | **0.24** | **(0.06–0.42)** |
| Health insurance  (ref. No) |  |  |  |  |  |  |  |  |  |  |  |  |
| Yes | **1.22** | **(1.10–1.34)** | 0.01 | (-0.00–0.02) | 0.02 | (-0.00–0.05) | 0.05 | (-0.00–0.09) | **0.09** | **(0.00–0.18)** | **0.14** | **(0.02–0.25)** |
| **Note:**  ^*^ All sample  ^†^ Respondents who reported at least one outpatient visits  ^‡^ PCE: Per capita expenditure. Costs were converted to 2014 International Dollars. Bootstrapping with 500 times replications was performed to estimate the standard error. Bold figure represents p-value <0.05 | | | | | | | | | | | | |

**Appendix 5. The incremental inpatient visits by population quintile**

| **Variables** | **Logistic regression (n=13,798)**^*^ | | **Quantile Regression**  **(n=630)**^†^ | | | | | | | | | |
| --- | --- | --- | --- | --- | --- | --- | --- | --- | --- | --- | --- | --- |
|  |  |  | 10^th^ percentile | | 25^th^ percentile | | 50^th^ percentile | | 75^th^ percentile | | 90^th^ percentile | |
|  | OR | (95% CI) | Coef. | (95% CI) | Coef. | (95% CI) | Coef. | (95% CI) | Coef. | (95% CI) | Coef. | (95% CI) |
| Number of NCDs  (ref. no NCD) |  |  |  |  |  |  |  |  |  |  |  |  |
| One | **1.90** | **(1.45–2.49)** | -0.00 | (-0.02–0.02) | 0.00 | (-0.03–0.04) | 0.01 | (-0.06–0.07) | -0.00 | (-0.09–0.09) | -0.08 | (-0.46–0.31) |
| Two | **3.60** | **(2.69–4.83)** | 0.01 | (-0.01–0.04) | 0.04 | (-0.00–0.09) | **0.09** | **(0.01–0.16)** | **0.18** | **(0.02–0.33)** | 0.33 | (-0.11–0.78) |
| Three or more | **6.67** | **(4.92–9.05)** | **0.04** | **(0.00–0.07)** | **0.09** | **(0.03–0.14)** | **0.16** | **(0.07–0.25)** | **0.38** | **(0.19–0.57)** | **0.46** | **(0.05–0.88)** |
| Age (year)  (ref. 40 – 49 years) |  |  |  |  |  |  |  |  |  |  |  |  |
| 50 – 59 years | 1.08 | (0.86–1.37) | -0.00 | (-0.02–0.02) | -0.00 | (-0.04–0.04) | -0.00 | (-0.07–0.06) | -0.01 | (-0.12–0.11) | -0.02 | (-0.36–0.31) |
| 60 – 69 years | **1.40** | **(1.08–1.82)** | 0.00 | (-0.02–0.03) | 0.00 | (-0.04–0.05) | 0.01 | (-0.07–0.08) | -0.02 | (-0.13–0.09) | -0.15 | (-0.47–0.18) |
| 70+ years | **1.88** | **(1.40–2.53)** | 0.00 | (-0.03–0.03) | 0.00 | (-0.05–0.05) | 0.01 | (-0.08–0.09) | 0.01 | (-0.14–0.17) | -0.12 | (-0.45–0.20) |
| Gender  (ref. Female) |  |  |  |  |  |  |  |  |  |  |  |  |
| Male | **1.32** | **(1.09–1.60)** | 0.01 | (-0.01–0.03) | 0.02 | (-0.02–0.05) | 0.04 | (-0.02–0.09) | 0.05 | (-0.05–0.14) | 0.05 | (-0.17–0.27) |
| Marital status  (ref. Not currently married) |  |  |  |  |  |  |  |  |  |  |  |  |
| Currently married | 1.03 | (0.81–1.30) | -0.00 | (-0.03–0.02) | -0.00 | (-0.04–0.04) | -0.00 | (-0.07–0.07) | -0.05 | (-0.18–0.09) | -0.22 | (-0.57–0.13) |
| Education status  (ref. No education) |  |  |  |  |  |  |  |  |  |  |  |  |
| Primary | 1.24 | (0.97–1.58) | -0.01 | (-0.03–0.02) | -0.02 | (-0.06–0.02) | -0.04 | (-0.10–0.03) | -0.06 | (-0.18–0.06) | -0.08 | (-0.43–0.26) |
| Junior high school | 0.92 | (0.66–1.28) | 0.00 | (-0.03–0.03) | -0.01 | (-0.07–0.05) | -0.02 | (-0.12–0.08) | -0.01 | (-0.17–0.15) | 0.20 | (-0.44–0.84) |
| Senior high school | 0.92 | (0.68–1.25) | -0.01 | (-0.04–0.02) | -0.03 | (-0.07–0.02) | -0.05 | (-0.12–0.03) | -0.07 | (-0.21–0.06) | -0.20 | (-0.58–0.18) |
| Tertiary | 0.71 | (0.49–1.04) | 0.00 | (-0.03–0.04) | -0.00 | (-0.07–0.07) | -0.01 | (-0.12–0.10) | -0.05 | (-0.21–0.12) | -0.33 | (-0.69–0.03) |
| Residency  (ref. Rural) |  |  |  |  |  |  |  |  |  |  |  |  |
| Urban | 0.98 | (0.80–1.20) | 0.01 | (-0.01–0.03) | 0.02 | (-0.01–0.05) | 0.04 | (-0.01–0.09) | 0.07 | (-0.02–0.16) | 0.22 | (-0.00–0.44) |
| Region  (ref. Java-Bali) |  |  |  |  |  |  |  |  |  |  |  |  |
| Sumatra | 0.98 | (0.78–1.22) | 0.01 | (-0.01–0.03) | 0.02 | (-0.02–0.07) | 0.04 | (-0.03–0.12) | 0.09 | (-0.04–0.22) | 0.22 | (-0.12–0.56) |
| Nusa Tenggara | 1.15 | (0.80–1.66) | 0.02 | (-0.03–0.06) | 0.03 | (-0.04–0.09) | 0.03 | (-0.08–0.14) | 0.04 | (-0.16–0.24) | 0.57 | (-0.17–1.32) |
| Kalimantan | 1.16 | (0.78–1.72) | 0.03 | (-0.03–0.08) | 0.04 | (-0.06–0.13) | 0.05 | (-0.10–0.20) | 0.13 | (-0.34–0.61) | 0.76 | (-0.91–2.44) |
| Sulawesi | 1.17 | (0.82–1.69) | 0.02 | (-0.03–0.06) | 0.03 | (-0.04–0.11) | 0.05 | (-0.07–0.17) | 0.10 | (-0.10–0.29) | 0.12 | (-0.23–0.47) |
| PCE^‡^ (ref. Q1) |  |  |  |  |  |  |  |  |  |  |  |  |
| Q2 | 0.99 | (0.72–1.37) | -0.01 | (-0.04–0.02) | -0.02 | (-0.08–0.04) | -0.03 | (-0.13–0.07) | -0.05 | (-0.22–0.12) | 0.08 | (-0.40–0.55) |
| Q3 | 1.36 | (0.99–1.85) | -0.02 | (-0.05–0.02) | -0.03 | (-0.09–0.03) | -0.06 | (-0.15–0.03) | -0.12 | (-0.27–0.03) | -0.13 | (-0.48–0.22) |
| Q4 | **1.52** | **(1.12–2.06)** | -0.01 | (-0.04–0.02) | -0.02 | (-0.07–0.04) | -0.04 | (-0.13–0.05) | -0.08 | (-0.23–0.07) | 0.07 | (-0.40–0.54) |
| Q5 | **2.00** | **(1.46–2.72)** | -0.01 | (-0.04–0.02) | -0.01 | (-0.07–0.04) | -0.03 | (-0.12–0.06) | -0.06 | (-0.21–0.09) | 0.09 | (-0.37–0.55) |
| Health insurance  (ref. No) |  |  |  |  |  |  |  |  |  |  |  |  |
| Yes | **2.17** | **(1.78–2.64)** | -0.01 | (-0.03–0.01) | -0.02 | (-0.05–0.02) | -0.03 | (-0.09–0.03) | -0.05 | (-0.14–0.04) | -0.09 | (-0.38–0.20) |
| **Note:**  ^*^ All sample  ^†^ Respondents who reported at least one inpatient visits  ^‡^ PCE: Per capita expenditure. Costs were converted to 2014 International Dollars. Bootstrapping with 500 times replications was performed to estimate the standard error. Bold figure represents p-value <0.05 | | | | | | | | | | | | |

**Appendix 6. The incremental out-of-pocket expenditure by population quintile**

| **Variables** | **Logistic regression (n=13,798)**^*^ | | **Quantile regression**  **(n=2,097)**^†^ | | | | | | | | | |
| --- | --- | --- | --- | --- | --- | --- | --- | --- | --- | --- | --- | --- |
|  |  |  | 10^th^ percentile | | 25^th^ percentile | | 50^th^ percentile | | 75^th^ percentile | | 90^th^ percentile | |
|  | OR | (95% CI) | Coef. | (95% CI) | Coef. | (95% CI) | Coef. | (95% CI) | Coef. | (95% CI) | Coef. | (95% CI) |
| Number of NCDs  (ref. no NCD) |  |  |  |  |  |  |  |  |  |  |  |  |
| One | **1.51** | **(1.32–1.71)** | 0.51 | (-0.30–1.32) | 0.45 | (-0.34–1.23) | 0.81 | (-0.82–2.45) | 3.68 | (-2.21–9.57) | 10.73 | (-4.57–26.02) |
| Two | **2.39** | **(2.04–2.80)** | **1.44** | **(0.32–2.55)** | **2.80** | **(1.12–4.47)** | **5.95** | **(2.93–8.97)** | **14.55** | **(3.76–25.34)** | 30.55 | (-0.61–61.71) |
| Three or more | **3.55** | **(2.96–4.27)** | 0.30 | (-0.98–1.58) | **2.61** | **(0.40–4.81)** | **10.12** | **(4.90–15.35)** | **25.08** | **(10.18–39.98)** | 110.6 | (39.13–182.13) |
| Age (year)  (ref. 40 – 49 years) |  |  |  |  |  |  |  |  |  |  |  |  |
| 50 – 59 years | 1.05 | (0.93–1.20) | 0.48 | (-0.47–1.43) | **1.08** | **(0.10–2.06)** | **2.59** | **(0.69–4.50)** | 2.92 | (-3.36–9.20) | 4.10 | (-13.84–22.04) |
| 60 – 69 years | 1.02 | (0.87–1.20) | 0.43 | (-0.60–1.46) | 0.23 | (-1.01–1.47) | 1.48 | (-0.76–3.71) | 4.30 | (-3.71–12.31) | 7.91 | (-17.18–33.00) |
| 70+ years | **0.68** | **(0.55–0.84)** | 1.04 | (-0.45–2.52) | **1.72** | **(0.06–3.37)** | **3.26** | **(0.08–6.44)** | **13.98** | **(0.73–27.23)** | 16.92 | (-28.51–62.36) |
| Gender  (ref. Female) |  |  |  |  |  |  |  |  |  |  |  |  |
| Male | **1.51** | **(1.35–1.69)** | 0.93 | (0.13–1.73) | 0.83 | (-0.02–1.67) | 1.12 | (-0.68–2.91) | 1.80 | (-4.96–8.55) | 29.82 | (-0.25–59.88) |
| Marital status  (ref. Not currently married) |  |  |  |  |  |  |  |  |  |  |  |  |
| Currently married | **1.31** | **(1.13–1.51)** | 0.81 | (-0.15–1.77) | 0.70 | (-0.60–2.00) | 0.86 | (-1.47–3.20) | 5.96 | (-0.42–12.33) | 7.09 | (-8.90–23.09) |
| Education status  (ref. No education) |  |  |  |  |  |  |  |  |  |  |  |  |
| Primary | 0.99 | (0.86–1.14) | 0.14 | (-0.77–1.04) | -0.38 | (-1.41–0.65) | -0.66 | (-2.68–1.35) | 1.78 | (-4.48–8.04) | 1.10 | (-16.50–18.70) |
| Junior high school | 0.94 | (0.78–1.14) | -0.28 | (-1.68–1.11) | -0.32 | (-1.67–1.03) | 0.10 | (-2.91–3.11) | 3.26 | (-8.53–15.04) | **95.24** | **(7.85–182.62)** |
| Senior high school | 0.88 | (0.74–1.05) | 0.59 | (-0.82–2.00) | 0.57 | (-0.70–1.84) | 0.56 | (-2.38–3.49) | 6.15 | (-9.91–22.22) | **43.51** | **(3.50–83.52)** |
| Tertiary | 0.81 | (0.64–1.02) | **1.58** | **(0.24–2.91)** | 0.89 | (-1.02–2.80) | 0.81 | (-4.16–5.78) | 4.97 | (-16.64–26.59) | 71.00 | (-14.25–156.25) |
| Residency  (ref. Rural) |  |  |  |  |  |  |  |  |  |  |  |  |
| Urban | **0.81** | **(0.73–0.91)** | -0.11 | (-0.87–0.65) | 0.45 | (-0.37–1.26) | 0.41 | (-1.29–2.11) | 3.29 | (-2.33–8.91) | -8.75 | (-23.87–6.38) |
| Region  (ref. Java-Bali) |  |  |  |  |  |  |  |  |  |  |  |  |
| Sumatra | **0.81** | **(0.71–0.92)** | 0.00 | (-0.82–0.82) | -0.13 | (-1.15–0.90) | 0.46 | (-1.53–2.45) | 0.81 | (-7.87–9.49) | 4.41 | (-22.87–31.70) |
| Nusa Tenggara | 0.96 | (0.77–1.18) | -1.16 | (-2.55–0.22) | -1.02 | (-2.55–0.51) | -1.48 | (-4.14–1.19) | 2.70 | (-8.70–14.10) | 13.88 | (-25.35–53.11) |
| Kalimantan | **0.69** | **(0.54–0.88)** | -0.23 | (-2.01–1.56) | -1.27 | (-3.06–0.52) | -2.03 | (-6.26–2.19) | -7.31 | (-24.52–9.90) | -24.26 | (-145.82–97.29) |
| Sulawesi | **0.41** | **(0.31–0.54)** | 0.97 | (-1.01–2.94) | 0.00 | (-2.88–2.88) | 0.41 | (-10.3–11.1) | 22.54 | (-6.17–51.25) | -2.14 | (-191.70–187.41) |
| PCE^‡^ (ref. Q1) |  |  |  |  |  |  |  |  |  |  |  |  |
| Q2 | **1.44** | **(1.19–1.73)** | **1.83** | **(0.65–3.01)** | 1.02 | (-0.17–2.21) | 1.53 | (-0.51–3.56) | 4.70 | (-0.88–10.29) | 2.98 | (-13.58–19.55) |
| Q3 | **1.90** | **(1.58–2.27)** | **2.54** | **(1.53–3.56)** | **1.72** | **(0.48–2.95)** | **3.26** | **(0.74–5.77)** | **11.78** | **(4.04–19.52)** | **26.91** | **(2.87–50.95)** |
| Q4 | **2.10** | **(1.75–2.51)** | **2.01** | **(0.97–3.05)** | **1.84** | **(0.42–3.27)** | **3.97** | **(1.87–6.06)** | **9.95** | **(2.45–17.45)** | **22.72** | **(0.66–44.78)** |
| Q5 | **2.18** | **(1.81–2.62)** | **3.73** | **(2.63–4.83)** | **3.88** | **(2.28–5.47)** | **9.00** | **(5.42–12.59)** | **39.92** | **(23.66–56.18)** | **109.66** | **(59.84–159.47)** |
| Health insurance  (ref. No) |  |  |  |  |  |  |  |  |  |  |  |  |
| Yes | **0.84** | **(0.76–0.94)** | 0.37 | (-0.43–1.17) | -0.19 | (-0.98–0.60) | 0.97 | (-0.73–2.66) | 4.48 | (-1.63–10.59) | 4.83 | (-12.30–21.96) |
| **Note:**  ^*^ All sample  **^†^** Respondents who reported OOPE for outpatient/inpatient care  **^‡^** PCE: Per capita expenditure. Costs were converted to 2014 International Dollars. Bootstrapping with 500 times replications was performed to estimate the standard error. Bold figure represents p-value <0.05 | | | | | | | | | | | | |

**Appendix 7. Predictive four-weekly OOPE by number of NCDs**

Notes:

CI — confidence interval–NCDs — noncommunicable diseases

Respondents who had OOPE (OOPE ≥ Int$1) = 2,097

Bootstrapping with 500 times replications was performed to estimate the standard error.

Shaded area in the graph represents the confidence interval.

**Appendix 8. Average number of outpatient visit, inpatient visits, and four-weekly OOPE by population quintile**

| Variables | Outcomes by quantile | | | | | | | | | |
| --- | --- | --- | --- | --- | --- | --- | --- | --- | --- | --- |
|  | 10^th^ percentile | | 25^th^ percentile | | 50^th^ percentile | | 75^th^ percentile | | 90^th^ percentile | |
|  | Mean | (95% CI) | Mean | (95% CI) | Mean | (95% CI) | Mean | (95% CI) | Mean | (95% CI) |
| Times of outpatient care (n=2,894) | 0.07 | (0.07–0.08) | 0.17 | (0.16–0.18) | 0.31 | (0.29–0.33) | 0.70 | (0.67–0.73) | 1.20 | (1.14–1.25) |
| Times of inpatient care (n=630) | 0.02 | (0.02–0.03) | 0.06 | (0.05–0.08) | 0.12 | (0.10–0.15) | 0.20 | (0.14–0.21) | 0.64 | (0.56–0.75) |
| Average four-weekly OOPE ($Int) (n=2,097) | 3.8 | (3.4–4.3) | 7.6 | (6.5–8.7) | 12.7 | (12.4–13.0) | 38.1 | (34.2–42.1) | 101.0 | (88.3–115.0) |
| OOPE — out-of-pocket expenditure | | | | | | | | | | |
